# Dr. Bartley’s Case of Paralysis of the Muscles of the Face, Treated by Iodide of Potassium

**Published:** 1842-04-30

**Authors:** R. T. H. Bartley

**Affiliations:** Under Graduate of the University of London


*Paralysis of the muscles of the Face.* By R. T. H. Bartley, M.R.C.S., Under Graduate of the University of London.—Mary Morris, aged 30, laundress, came under my care with paralysis of the muscles of the left half of the face. The mouth was drawn to the right side; the power of winking the left eye, as in the case of Dr. Zabriskie, was suspended; and when told to frown, or elevate the eyebrows, the right half of the forehead was alone corrugated, giving to the countenance a most peculiar and half idiotic expression. The platysma of the right side was frequently spasmodically contracted on her attempting to speak; but this, I think, was a semi-voluntary act, occasioned by her feeling a want of power in the antagonist muscle. She complained of no headache; sensation everywhere perfect; and the motions of those organs and muscles about the jaw and face, supplied by the opthalmic, lingual, and other nerves, such as the tongue and eyeball, masseter, pterygoid, and temporal muscles, were freely exercised, and equally well on both sides. She attributed her symptoms to a "*chill*, caught by sleeping on a damp pillow." Having had previous experience of the good effects of iodide of potassium in my own person (having been similarly attacked, and from a similar cause, in the summer of 1837,) I prescribed five grains of the salt to be taken in a wine-glassful of water, three times a day; and I ordered the unguentum potass. iodid. to be rubbed in under the ear, in the situation of the exit of the portio dura, night and morning. Though previously treated by an experienced practitioner for six weeks, by bleeding, leeching, blistering, and other antiphlogistic measures, without the smallest benefit, the first time I saw her (five days) after prescribing the iodide, &c., there was marked improvement, evidenced chiefly by the returning power of the corrugator supercilii. I was in attendance at intervals of three or four days for the next month, and was much gratified at the expiration of that period in finding my patient *perfectly* restored, and that, too, by a rigid perseverance in those means which in my own case had been so eminently successful.

Remarks.—The above cases were evidently examples of paralysis of the portio dura, confined in each case to but one trunk, and quite independent of any faulty condition of the cerebral nervous centre.

Sir Charles Bell was, I believe, the first to describe its strictly local character; but from the fact of its having been so imperfectly noted by late nosologists, I am inclined to think—admitting at the same time its rarity—that it has been too frequently considered as dependent on cerebral disorder, and that in many cases it has been merged into the other symptoms of general paralysis.—*Lond. Med. Gaz.* March 4th, 1842.

